# Effect of Dapagliflozin on Glycemic Variability in Patients with Type 2 Diabetes under Insulin Glargine Combined with Other Oral Hypoglycemic Drugs

**DOI:** 10.1155/2020/6666403

**Published:** 2020-11-24

**Authors:** Menghui Luo, Xiaocen Kong, Huiying Wang, Xiaofang Zhai, Tingting Cai, Bo Ding, Yun Hu, Ting Jing, Xiaofei Su, Huiqin Li, Jianhua Ma

**Affiliations:** Department of Endocrinology, Nanjing First Hospital, Nanjing Medical University, Nanjing 210012, China

## Abstract

**Aim:**

To evaluate the effect of an inhibitor of sodium-glucose cotransporter 2 (SGLT-2 inhibitor, dapagliflozin) on glycemic variability in type 2 diabetes mellitus (T2D) under insulin glargine combined with oral hypoglycemic drugs, using a continuous glucose monitoring system (CGMS).

**Methods:**

This prospective, self-controlled, single-center clinical trial recruited 36 patients with T2D under combined insulin glargine and oral hypoglycemic drugs. General clinical data were collected. Fasting blood glucose (FBG), postprandial blood glucose (PBG), glycosylated hemoglobin (HbA1c), and C-peptide levels were assessed before and four weeks of dapagliflozin (10 mg per day) treatment. Blood glucose was monitored for 72 hours before and after treatment using CGMS.

**Results:**

After treatment with dapagliflozin, FBG decreased from 6.74 ± 1.78 to 5.95 ± 1.13 mmol/L (*p* < 0.05); PBG decreased from 13.04 ± 2.99 to 10.92 ± 3.26 mmol/L (*p* < 0.05); HbA1c decreased from 7.37 ± 0.96% to 6.94 ± 0.80%. The proportion of patients with HbA1c < 7% increased from 27.8% to 58.3%, and the proportion of patients with HbA1c < 7% and without level 2 hypoglycemia increased from 27.8% to 55.6% (*p* < 0.05). CGMS data showed reduction of the 24 h MBG, MAGE, time-above-range (TAR, >10 mmol/L), high blood glucose index (HBGI), glucose management indicator (GMI), and incremental area under the curve of the glucose level more than 10 mmol/L (AUC > 10) and an increase of time-in-range (TIR, 3.9-10 mmol/L) with treatment. Homeostasis model assessment for pancreatic beta-cell function (HOMA-beta) increased significantly with treatment (*p* < 0.05), and fewer insulin doses were required after the treatment, without increasing in hypoglycemia and urinary tract infection. Further, a stratified analysis showed that patients with higher pretreatment HbA1c and waist-to-hip ratio (WHR) had greater improvement in glycemic control.

**Conclusion:**

Dapagliflozin may reduce blood glucose levels, ameliorate glycemic variability, and improve pancreatic beta-cell function in patients with T2D under insulin glargine combined with other oral hypoglycemic drugs, especially in those with poor glucose control and abdominal obesity.

## 1. Introduction

Type 2 diabetes mellitus (T2D) is a metabolic disease characterized by chronic hyperglycemia and has become one of the common chronic diseases worldwide, especially in developing countries. In China, 10.9% of adults have diabetes, and the prediabetes prevalence rate is as high as 35.7% in China [1]. It is known that glycosylated hemoglobin (HbA1c) is closely associated with microvascular complications and cardiovascular disease outcomes [2]. More recently, evidence suggested that there are close relationships between glycemic variability and oxidative stress, endothelial dysfunction, and atherosclerosis [3–5]. Furthermore, glycemic variability is a potential risk factor for complications in patients with diabetes [6, 7] and is a predictor of cardiovascular complications [8]. Dapagliflozin, a selective inhibitor of sodium-glucose cotransporter 2 (SGLT-2), mainly reduces blood glucose by increasing urinary glucose excretion. Recent studies show that dapagliflozin combined with other hypoglycemic drugs with or without insulin treatment can reduce blood glucose levels and improve HbA1c levels [9, 10]. And some studies also mentioned that SGLT-2 inhibitors can improve glycemic variability in T2D receiving insulin treatment [11–14]. However, it is unclear that SGLT-2 inhibitors are more effective for which groups of people. Therefore, we investigated the effect of dapagliflozin in T2D under insulin glargine combined with other oral hypoglycemic drugs using a CGMS to evaluate glucose fluctuations and find out the most suitable one.

## 2. Methods

### 2.1. Study Participants

This study was approved by the Ethical Committee of Nanjing First Hospital, Nanjing Medical University in Nanjing, China. Informed consent was obtained from all patients.

The inclusion criteria were as follows: (1) a diagnosis of T2D, as defined by the World Health Organization criteria published in 1999, (2) age ≥ 18 years, (3) receiving a stable insulin glargine doses combined with oral hypoglycemic drugs for more than 3 months prior to screening, and (4) estimated glomerular filtration rate (eGFR) ≥ 60 mL/min/1.73m^2^. The main exclusion criteria were as follows: (1) type 1 diabetes, (2) severe hypoglycemic events or diabetic ketoacidosis (DKA) within 6 months prior to screening, (3) pregnant women, (4) cardiovascular or cerebrovascular accident ≤ 12 weeks before screening, and (5) acute and chronic severe infectious diseases.

### 2.2. Study Protocol

A prospective, self-controlled, single-center clinical trial was conducted (NCT03631134). A total of 36 patients with T2D (21 men and 15 women) were enrolled from June 2017 to June 2019. Eligible patients received dapagliflozin (10 mg per day) for 4 weeks during the treatment period. Patients were asked to continue their previous treatment regimen and maintain moderate physical activity and diet. If blood glucose was less than 4.4 mmol/L by self-monitoring or the patient had symptoms of hypoglycemia, the patient was asked to reduce insulin doses. They were instructed to ingest food when blood glucose level was less than 3.9 mmol/L by self-monitoring or when they had symptoms of hypoglycemia.

Demographic and clinical data were collected by the same person throughout the study, including medical history and medicine use. Anthropometric parameters, such as height, body weight, hip circumference, and waist circumference, were measured before and after the 4-week treatment. Similarly, biochemical parameters were measured before and after treatment, including fasting blood glucose (FBG), postprandial blood glucose (PBG), alanine aminotransferase (ALT), serum creatinine (SCr), total cholesterol (TC), glycosylated hemoglobin (HbA1c), and C-peptide levels. The eGFR was calculated as follows: eGFR (mL/min/1.73m^2^) = 186 × (SCr/88.4)^−1.154^ × (age)^−0.203^ × (0.742 if female). The homeostasis model assessment for pancreatic beta-cell function (HOMA-beta) was calculated as follows: HOMA‐beta = 270 × (fasting C − peptide)/[0.333 × (FBG − 3.5)]. The homeostasis model assessment of C-peptide secretion (HOMA-CR) was calculated as follows: HOMA‐CR = 1.5 + FBG × (fasting C − peptide)/(2.8 × 0.333). A standard meal test was designed by a specialist researcher, which included 87.9% of carbohydrate, 8.8% of protein, and 3.3% of fat. A CGMS (Medtronic MiniMed, USA) was used to monitor blood glucose every 5 minutes for 3 days before the study and on days 26-28. Data from 0:00 to 24:00 day 2 of CGMS was to analyze the glycemic profile. In detail, the time-in-range (TIR, 3.9-10 mmol/L), 24-hour mean blood glucose (24 h MBG), 24-hour mean amplitude of glycemic excursion (MAGE), the incremental area under the curve of the glucose level (AUC), high blood glucose index (HBGI), low blood glucose index (LBGI), and glucose management indicator (GMI) were calculated.

### 2.3. Statistical Analysis

Statistical analyses were performed using SPSS version 20.0 (IBM Corp., Armonk, NY). Normally distributed data are presented as the mean ± standard deviation (SD), and nonnormally distributed data are presented as median (25th–75th range). The paired *t*-test and Wilcoxon test were used to evaluate differences in glycemic profile before and after treatment. The independent-samples *t*-test and Mann-Whitney *U* test were used to evaluate differences in glycemic profile between patients who did and did not achieve HbA1c ≥ 7% with treatment. The one-way analysis of variance (ANOVA) and *K*-independent samples test were used to evaluate differences in pretreatment characteristics among tertiles of the treatment-related reduction in the HbA1c level. The chi-square test was used to compare qualitative data. The accepted level of significance was 0.05, using two-tailed tests.

## 3. Results

### 3.1. Baseline Characteristics

The baseline characteristics of the participants are shown in Table 1. The average duration of diabetes was 10.92 ± 4.92 years, and the average pretreatment HbA1c level was 7.37 ± 0.96%. The pretreatment HbA1c level was higher than 7% in 72.2% of patients.

### 3.2. The 24 h Glycemic Profile

**Figure 1:**
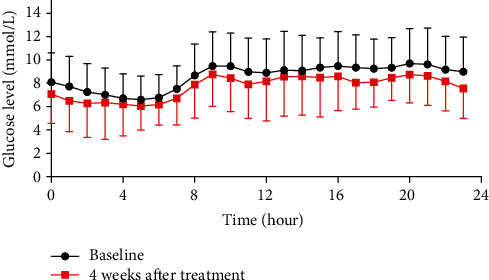
24 h CGMS glucose profile of patients.

The 24 h CGMS glucose profile of patients is shown in Figure 1. The 24 h MBG, MAGE, HBGI, GMI, the incremental area under the curve of the glucose level more than 10 mmol/L (AUC > 10), and time-above-range (TAR, >10 mmol/L) were reduced, and the TIR was increased, after the 4-week treatment compared to pretreatment values (*p* < 0.05). However, there was no difference in the time-below-range (TBR, <3.9 mmol/L) (Table 2).

Additionally, patients who achieved HbA1c ≥ 7% after treatment showed obvious improvement in 24 h MBG, TAR, AUC3.9-10, HBGI, GMI, and AUC > 10 (*p* < 0.05) (Table 3).

### 3.3. Glycemic Control and Pancreatic Beta-Cell Function

Compared to pretreatment values, patients required fewer daily insulin doses after treatment for a 4-week treatment period (*p* < 0.05). The homeostasis model assessment for pancreatic beta-cell function (HOMA-beta) increased significantly after treatment. Additionally, FBG, PBG, and HbA1c levels after the standard meal test were lower after treatment compared to pretreatment values (Table 4). The proportion of patients who achieved HbA1c < 7% increased from 27.8% to 58.3% (*p* < 0.05). Additionally, the proportion of patients who achieved HbA1c < 7% without level 2 hypoglycemia (glucose concentration < 3.0 mmol/L) increased from 27.8% to 55.6% (*p* < 0.05).

In order to further explore the characteristics of patients with a greater decrease in the HbA1c level, the baseline data were analyzed in the terms of tertiles in the treatment-related reduction in the HbA1c level. High, mid, and low degree of treatment-related reduction in the HbA1c level were defined as reductions ≥ 0.6%, 0.3-0.5%, and ≤0.2%, respectively. We found that patients with a higher pretreatment waist-to-hip ratio and HbA1c level had a greater treatment-related decrease in the HbA1c level (Table 5).

### 3.4. Hypoglycemia and Urinary Tract Infection

Level 3 hypoglycemia (defined as a severe event characterized by altered mental and/or physical status requiring assistance for treatment of hypoglycemia) did not occur during the study period. There was no change in the rates of level 1 hypoglycemia (glucose concentration < 3.9 mmol/L and ≥3.0 mmol/L) and level 2 hypoglycemia (glucose concentration < 3.0 mmol/L) with treatment (*p* > 0.05). Furthermore, the incidence of urinary tract infection did not change with treatment.

## 4. Discussion

The present study showed that patients under insulin glargine combined with oral hypoglycemic drugs benefited from dapagliflozin treatment in terms of improvements in the mean glucose level and glycemic variability, without an increase in the incidence of hypoglycemia and urinary tract infection. Patients with higher HbA1c and waist-to-hip ratio before treatment were more beneficial in improving HbA1c. Another important outcome from the present study was that the SGLT-2 inhibitor, dapagliflozin, was able to partially replace exogenous insulin and improve pancreatic beta-cell function.

The HbA1c is used to assess glycemic control in diabetes mellitus during three months as a gold standard. Many large studies showed a higher mean HbA1c level usually companies with a higher incidence of diabetic complications, even can predict and partly explain cardiovascular disease [15–17]. However, the HbA1c level is affected by individual factors, including genetic, hematological conditions, and ethnicity [18–20]. More importantly, the HbA1c cannot reflect glycemic variability. It was reported that glycemic variability still is an independent risk of diabetic neuropathy, although at the same mean glucose level [21]. And glycemic variability has a greater and worse effect on cardiovascular disease than HbA1c [22]. Thus, It was not enough to rely only on HbA1c to guide glycemic management. Recently, some studies have focused on glycemic variability as another metric for glycemic control. The relationship between diabetes-related complications and glycemic variability may result from cardiovascular damage and hypoglycemia [23]. CGMSs utilize a monitoring technology that indirectly detects the blood glucose level in the interstitial fluid via a sensor every 5 minutes. This provides a complete and available glycemic profile and enables us to a better understanding of the glycemic variability and detection of hypoglycemia in time.

The American Diabetes Association emphasizes the importance of SGLT-2 inhibitors as combination therapy, especially in patients with poor glucose control and atherosclerotic cardiovascular disease [24]. It was reported that HbA1c and FPG levels showed greater reductions with 52 weeks of dapagliflozin treatment, compared to that with placebo [25]. Similarly, our study suggested that FBG, PBG, and HbA1c reduced with dapagliflozin treatment. Further, we found that patients with abdominal obesity and poor glycemic control might be more suitable for dapagliflozin treatment.

A previous study showed that dapagliflozin had a beneficial effect on glycemic variability in T2D under either insulin or metformin [26]. Another study indicated that dapagliflozin also has a positive effect on glycemic control, without the occurrence of hypoglycemia, in patients with type 1 diabetes [27]. This study explored that dapagliflozin was used as monotherapy or as a supplement to other oral hypoglycemic drugs or insulin for diabetic treatment. Our study also showed dapagliflozin improved glycemic variability without increasing the incidence of hypoglycemia. Furthermore, we analyzed the differences in the glycemic profile of patients with different HbA1c levels, in order to find out that dapagliflozin was more suitable for which kind of population in T2D under insulin glargine combined with other oral hypoglycemic drugs. As a result, using CGM data, we found that patients with higher HbA1c levels showed shorter duration of euglycemia, longer duration of hyperglycemia, greater glycemic variability, and greater improvement in hyperglycemia and euglycemia than patients with lower levels of HbA1c.

SGLT2 inhibitors can increase insulin secretion and beta-cell mass but cannot increase insulin sensitivity [28–30]. However, Xu el. [31] reported that mice who were fed a high-fat diet showed amelioration of insulin resistance, via an increase in insulin receptors, after SGLT-2 inhibitor treatment. However, there are few studies to evaluate the effect of SGLT-2 inhibitors combined with insulin glargine and other oral hypoglycemic drugs on pancreatic beta-cell in T2D. We focused on dapagliflozin as a basal insulin supplement therapy with T2D and showed that dapagliflozin played an important role in improving pancreatic beta-cells and reducing insulin doses.

The present study has some limitations. Its sample size was small. In the future, we intend to increase the number of samples to further confirm our conclusions. And the study period was short; therefore, it is unclear whether glycemic variability improved by dapagliflozin can contribute to long-term benefit from T2D under insulin glargine combined with other oral hypoglycemic drugs. We need to extend the research time to confirm it. Our study has no control group; it may be difficult to exclude the placebo effect of patients after taking medicine.

In summary, patients with T2D under insulin glargine combined with oral hypoglycemic drugs had lower HbA1c levels and glycemic variability and better beta-cell function, consistent with a reduced need for insulin doses, especially in patients with higher pretreatment HbA1c levels, after dapagliflozin treatment. More importantly, patients with poor glucose control and abdominal obesity had greater benefits with dapagliflozin treatment.

## Figures and Tables

**Table 1:** Patient baseline characteristics.

Characteristics	Whole group (*n* = 36)
Age (years)	58.33 ± 9.50
Sex (M/F)	21/15
Diabetes duration (years)	10.92 ± 4.92
Insulin dose (IU/d)	21.67 ± 7.89
BMI (kg/m^2^)	25.22 ± 3.21
Body weight (kg)	69.28 ± 11.43
Waist-to-hip radio	0.94 ± 0.05
SBP (mmHg)	135.86 ± 15.23
DBP (mmHg)	84.47 ± 20.68
FBG (mmol/L)	6.74 ± 1.78
HbA1c (%)	7.37 ± 0.96
ALT (U/L)	24.53 ± 12.14
eGFR (mL/min/1.73 m^2^)	111.03 ± 19.59
TC (mmol/L)	4.51 ± 1.03
HOMA-beta	317.64 ± 241.91
HOMA-CR	9.89 ± 7.60
Oral hypoglycemic drugs (%)	
Secretagogues	50.0
Metformin	77.8
Thiazolidine	8.3
*α*-Glucosidase inhibitor	52.8
DPP4 inhibitor	5.6

BMI: body mass index; SBP: systolic blood pressure; DBP: diastolic blood pressure; FBG: fasting blood glucose; HbA1c: glycosylated hemoglobin; ALT: alanine aminotransferase; eGFR: estimated glomerular filtration rate; TC: total cholesterol; HOMA-beta: homeostasis model assessment for pancreatic beta-cell function; HOMA-CR: homeostasis model assessment of C-peptide secretion; DPP4: dipeptidase-4 inhibitor.

**Table 2:** 24 h glycemic profiles.

	Baseline	4 weeks after the treatment	*p*
24 h MBG (mmol/L)	8.56 ± 1.77	7.43 ± 1.03^∗^	0.001
MAGE (mmol/L)	4.80 ± 2.73	3.73 ± 2.10^∗^	0.048
TIR (%)	72.45 ± 26.19	85.56 ± 14.88^∗^	0.002
TAR (%)	20.66 (6.95, 40.80)	9.38 (0.00, 18.49)^∗^	0.001
TBR (%)	0.00 (0.00, 0.00)	0.00 (0.00, 1.39)	0.796
AUC3.9-10 (mmol/L∗d)	242.04 ± 61.11	199.14 ± 48.15^∗^	0.002
AUC > 10 (mmol/L∗d)	11.35 (2.64, 51.54)	5.78 (0.00, 20.51)^∗^	0.003
AUC < 3.9 (mmol/L∗d)	0.00 (0.00, 0.00)	0.00 (0.00, 0.00)	0.959
HBGL	6.08 ± 5.38	4.04 ± 2.83^∗^	0.000
LBGL	0.68 (0.00, 2.23)	1.18 (0.25, 2.68)	0.223
GMI (mmol/mol)	52.99 ± 8.32	47.68 ± 4.88^∗^	0.001

Data were shown as mean ± SD or median (first quartile, third quartile). ^∗^Baseline vs. 4 weeks after the treatment: *p* < 0.05. 24 h MBG: 24-hour mean blood glucose; MAGE: 24-hour mean amplitude of glycemic excursion; TIR: time-in-range (3.9-10 mmol/L); TAR: time-above-target range (>10 mmol/L); TBR: time-below-target ranges (<3.9 mmol/L); AUC3.9-10: the incremental area under the curve of the glucose level between 3.9 and 10 mmol/L; AUC > 10: the incremental area under the curve of the glucose level more than 10 mmol/L; AUC < 3.9: the incremental area under the curve of the glucose level less than 3.9 mmol/L; HBGI: high blood glucose index; LBGI: low blood glucose index; GMI: glucose management indicator.

**Table 3:** Dynamic blood glucose profile of patients with different HbA1c stratification before and after treatment.

	Baseline		4 weeks after treatment		*Δ*	
	High-A_1C_ (≥7%)	Low-A_1C_ (<7%)	High-A_1C_ (≥7%)	Low-A_1C_ (<7%)	High-A_1C_ (≥7%)	Low-A_1C_ (<7%)
24 h MBG	9.07 ± 1.72	7.23 ± 1.09^∗^	7.52 ± 1.10	7.21 ± 0.88	−1.55 ± 1.76	−0.02 ± 1.48^∗^
MAGE	5.60 ± 2.66	2.71 ± 1.67^∗^	3.97 ± 2.16	3.11 ± 1.87	−1.63 ± 3.17	0.39 ± 2.66
TIR (%)	65.10 ± 26.35	91.56 ± 13.32^∗∗^	82.32 ± 15.71	93.99 ± 8.12^∗^	17.22 ± 24.27	2.43 ± 12.95
TAR (%)	24.83 (12.76, 43.15)	0.00 (0.00, 16.93)^∗^	9.72 (0.00, 25.70)	0.70 (0.00, 12.76)	-17.54 (-29.08, -3.38)	0.52 (-4.17, 3.22)^∗^
TBR (%)	0.00 (0.00, 1.74)	0.00 (0.00, 0.00)	0.00 (0.00, 1.65)	0.00 (0.00, 0.35)	0.00 (-1.74, 1.39)	0.00 (0.00, 0.35)
AUC3.9-10	260.22 ± 51.92	194.75 ± 59.95^∗^	200.91 ± 48.03	194.54 ± 50.77	−59.32 ± 69.79	−0.21 ± 83.29^∗^
AUC > 10	26.48 (5.41, 70.53)	0.00 (0.00, 9.32)^∗∗^	6.89 (0.00, 28.58)	0.10 (0.00, 7.03)	-12.37 (-52.96, 0.15)	0.00 (-6.57, 5.20)^∗^
AUC < 3.9	0.00 (0.00, 0.47)	0.00 (0.00, 0.00)	0.00 (0.00, 0.23)	0.00 (0.00, 0.02)	0.00 (-0.47, 0.11)	0.00 (0.00, 0.02)
HBGI	8.33 ± 5.42	2.84 ± 2.59^∗∗^	4.57 ± 3.00	2.64 ± 1.79	−3.75 ± 4.13	−0.20 ± 2.68^∗^
LBGI	0.45 (0.01, 3.03)	0.89 (0.00, 1.57)	1.30 (0.39, 3.24)	0.51 (0.19, 1.64)	0.78 ± 3.42	−0.05 ± 1.58
GMI	55.41 ± 8.10	46.72 ± 5.13^∗∗^	48.10 ± 5.15	46.61 ± 4.14	−7.31 ± 8.28	−0.11 ± 6.98^∗^

^∗^ *p* < 0.05; ^∗∗^*p* < 0.01. 24 h MBG: 24-hour mean blood glucose; MAGE: 24-hour mean amplitude of glycemic excursion; TIR: time-in-range (3.9-10 mmol/L); TAR: time-above-target range (>10 mmol/L); TBR: time-below-target ranges (<3.9 mmol/L); AUC3.9-10: the incremental area under the curve of the glucose level between 3.9 and 10 mmol/L; AUC > 10: the incremental area under the curve of the glucose level more than 10 mmol/L; AUC < 3.9: the incremental area under the curve of the glucose level less than 3.9 mmol/L; HBGI: high blood glucose index; LBGI: low blood glucose index; GMI: glucose management indicator.

**Table 4:** Glycemic control and pancreatic *β*-cell function.

	Baseline	4 weeks after treatment	*p*
Insulin dose (IU/d)	21.67 ± 7.89	18.28 ± 7.52^∗^	0.000
HbA1c (%)	7.37 ± 0.96	6.94 ± 0.80^∗^	0.000
FBG (mmol/L)	6.74 ± 1.78	5.95 ± 1.13^∗^	0.015
PBG 30 min (mmol/L)	8.59 ± 1.85	7.59 ± 1.83^∗^	0.004
PBG 120 min (mmol/L)	13.04 ± 2.99	10.92 ± 3.26	0.005
C-peptide 0 min (ng/mL)	1.10 ± 0.88	1.15 ± 0.73	0.589
C-peptide 30 min (ng/mL)	1.62 ± 1.04	1.55 ± 0.87	0.372
C-peptide 120 min (ng/mL)	3.24 ± 2.08	3.51 ± 1.66	0.382
HOMA-CR	9.89 ± 7.60	9.30 ± 5.46	0.543
HOMA-beta	317.64 ± 241.91	412.51 ± 273.48^∗^	0.046

Data were shown as mean ± SD or median (first quartile, third quartile). ^∗^Baseline vs. 4 weeks after the treatment: *p* < 0.05. HbA1c: glycosylated hemoglobin; FBG: fasting blood glucose; PBG 30 min: postprandial blood glucose at 30 min after a standard meal; PBG 120 min: postprandial blood glucose at 120 min after a standard meal; C-peptide 0 min: C-peptide level at 0 min after a standard meal; C-peptide 30 min: C-peptide level at 30 min after a standard meal; C-peptide 120 min: C-peptide level at 120 min after a standard meal; HOMA-CR: homeostasis model assessment of C-peptide secretion; HOMA-beta: homeostasis model assessment for pancreatic beta-cell.

**Table 5:** Difference in baseline characteristics between different drops of HbA1c.

	High degree (≥0.6%)	Mid degree (0.3-0.5%)	Low degree (≤0.2%)	*p*
Age (years)	59.42 ± 8.40	52.67 ± 10.84	60.87 ± 8.63	0.108
Duration (years)	11.75 ± 4.75	11.33 ± 5.52	10.00 ± 4.87	0.641
Body weight (kg)	70.04 ± 10.04	73.17 ± 9.41	66.33 ± 13.31	0.362
BMI (kg/m^2^)	25.60 ± 3.50	26.23 ± 2.84	24.31 ± 3.14	0.331
Waist-to-hip ratio	0.96 ± 0.04	0.94 ± 0.04	0.91 ± 0.06	0.025
eGFR (mL/min^−1^/1.73 m^2^)	121.69 ± 10.64	107.47 ± 15.10	104.63 ± 24.31	0.086
HbA1c (%)	7.94 ± 1.19	7.32 ± 0.79	6.93 ± 0.58	0.029
Insulin dose (IU/d)	22.17 ± 8.80	24.44 ± 10.28	19.60 ± 4.95	0.606
HOMA-CR	8.40 ± 3.89	11.32 ± 5.06	10.15 ± 10.72	0.723
HOMA-beta	289.91 ± 224.45	265.75 ± 133.65	370.91 ± 304.27	0.584

Data were shown as mean ± SD or median (first quartile, third quartile). BMI: body mass index; eGFR: estimated glomerular filtration rate; HbA1c: glycosylated hemoglobin; HOMA-CR: homeostasis model assessment of C-peptide secretion; HOMA-beta: homeostasis model assessment for pancreatic beta-cell.

## Data Availability

All the data used to support the findings of this study are available from the corresponding author upon request.
